# Extracellular signal-regulated kinase 3 forms a nuclear complex with Aly/REF export factor and the splicing factor proline and glutamine rich to exacerbate pathological cardiac remodeling due to pressure overload

**DOI:** 10.1186/s43556-026-00563-9

**Published:** 2026-09-18

**Authors:** Wen-Ying Zhou, Feng Wang, Shu-Yu Wang, Li-Guo Wang, Ai-Qun Chen, Haoyue Tang, Ya-Peng Chen, Huan Zhang, Xiao-Fei Gao, Juan Zhang, Shao-Liang Chen, Jun-Jie Zhang

**Affiliations:** 1 Division of Cardiology, Nanjing First Hospital, Nanjing Medical University, 68 Changle Road, Nanjing, China; 2 Key Laboratory of Cardiovascular Intervention and Regenerative Medicine of Zhejiang Province, Department of Cardiology, School of Medicine, Sir Run-Run Shaw Hospital, Zhejiang University, Hangzhou, China

**Keywords:** Pathological cardiac remodeling, ERK3, ALY, Nuclear transportation, Estrone sulfate, Oxidative stress

## Abstract

Pathological cardiac remodeling is a significant contributor to heart failure and mortality. Studies have demonstrated that extracellular signal regulated kinase 3 (ERK3) in cardiac fibroblasts aggravates pressure overload-evoked pathological cardiac remodeling via mitogen activated protein kinase-activated protein kinase-5 (MK5). However, myocardial ERK3 does not activate MK5, thus the role of myocardial ERK3 remains unclear. Through in vivo and in vitro experiments, we revealed that myocardial ERK3 expression increases under pressure overload and the protein accumulates significantly in the nucleus. Using cardiomyocyte-specific *Erk3*-deficient mice, neonatal rat cardiomyocytes and adult mouse cardiomyocytes, we found that cardiomyocyte-specific *Erk3* deficiency ameliorated pressure overload-induced pathological cardiac remodeling in vivo. Co-immunoprecipitation, mass spectrometry, and single nucleus RNA sequencing were used to analyse for the nuclear translocation and downstream signaling mechanisms of ERK3. Specifically, pressure overload enhances the binding of Aly/REF export factor (ALY) to ERK3, resulting in the truncation of the ERK3 C-terminal and its subsequent nuclear translocation to activate the thioredoxin-interacting protein (TXNIP)/NOD-like receptor thermal protein domain associated protein 3 (NLRP3) pathway through the expression of the splicing factor proline and glutamine rich (SFPQ). To screen for targeted drugs, we conducted a virtual screening and identified estrone sulfate as an inhibitor of ALY to counteract hypertrophic effects. Collectively, our findings indicate that estrone sulfate functions as a novel inhibitor of the ALY-ERK3 signaling pathway, potentially serving as a promising therapeutic candidate for the management of pathological cardiac remodeling.

**Supplementary Information:**

The online version contains supplementary material available at https://doi.org/10.1186/s43556-026-00563-9.

## Introduction

Multiple factors such as aortic stenosis and hypertension increase the afterload on the heart, imposing mechanical stress on cardiac walls and triggering compensatory hypertrophy [1–3]. Although cardiac hypertrophy can temporarily preserve cardiac function, persistent pressure overload often leads to pathological cardiac remodeling, causing severe complications such as heart failure and sudden cardiac death [2]. Current therapies target both symptom alleviation and the inhibition of pathological cardiac remodeling, yet the underlying mechanisms remain incompletely understood. Consequently, a better understanding of the pathological mechanisms involved could facilitate the development of targeted therapies for pressure overload-induced cardiomyopathy.

Classic mitogen‐activated protein kinase (MAPKs), such as the extracellular signal regulated kinase 1/2 (ERK1/2) and p38 MAPK have been widely studied in the context of cardiomyopathy development [4–8]. In contrast, the role of atypical MAPKs, such as ERK3, in determining pathological cardiac remodeling remain unclear. Previous studies have shown that fibroblast ERK3 plays an important role in regulating cardiac fibroblast function through its interaction with MAPK-activated protein kinase-5 (MK5), as demonstrated by co-immunoprecipitation (CoIP), whereas ERK3 in cardiomyocytes does not have the same effect; thus the role of myocardial ERK3 is uncertain [9–12]. While ERK3 haplodeficiency alters pathological cardiac remodeling only upon afterload stress, the precise regulators of ERK3 activation are currently unknown [12].

Compared with conventional MAPKs, ERK3 has a distinctive structural feature: the presence of an elongated C-terminal tail that extends 180 amino acids beyond the conserved ERK core [13–15]. Researchers have reported that the nucleocytoplasmic shuttling of ERK3 is linked to the truncation of its C-terminal tail, which is required for its regulatory effect [16, 17]. Full-length ERK3 and ERK3 lack a C-terminal tail migrate at approximately 105 kDa and 62 kDa, respectively [16, 17]. However, the specific nuclear entry mechanism of ERK3 remains unclear. In this study, we explored the role of myocardial ERK3 in pathological cardiac remodeling and its underlying mechanisms. We found that pressure overload increases the expression of ERK3, particularly its nuclear accumulation. The accumulation of myocardial ERK3 in the nucleus may exacerbate pathological cardiac remodeling induced by pressure overload through the splicing factor proline and glutamine rich (SFPQ)/thioredoxin-interacting protein (TXNIP)/NOD-like receptor thermal protein domain associated protein 3 (NLRP3) pathway. These findings elucidate the role of myocardial ERK3 in pathological cardiac remodeling and hold promise for preventing and therapeutically intervening in pressure overload-induced pathological cardiac remodelling.

Additionally, we identified Aly/REF export factor (ALY) as a key executor that facilitates the nuclear transport of ERK3 by removing its C-terminal tail, and we explored estrone sulfate (ES) as a potential new ALY-targeted inhibitor. ALY serves as a significant nuclear export cofactor and is primarily engaged in the processing and transport of mRNA [18]. Our findings reveal a potential novel function of ALY in the regulation of protein nuclear transport. Moreover, the literature on the involvement of ALY in cardiovascular diseases is limited. This study presents novel evidence indicating that ALY may be capable of modulating stress-induced pathological cardiac remodeling.

Collectively, these findings elucidate the role of myocardial ERK3 in the context of pressure overload-induced pathological cardiac remodeling and the mechanisms underlying ERK3 activation. Through in vitro and in vivo experiments combined with sequencing, we revealed that the nuclear transport of ERK3 in cardiomyocytes may exacerbate pathological cardiac remodeling caused by pressure overload via the ALY-ERK3-SFPQ complex. Consequently, targeting the ALY-ERK3 complex may represent a promising therapeutic strategy for the management of pathological cardiac remodeling and heart failure.

## Results

### Myocardial ERK3 accumulation evoked by pressure overload is more pronounced in the nucleus

**Fig. 1:**
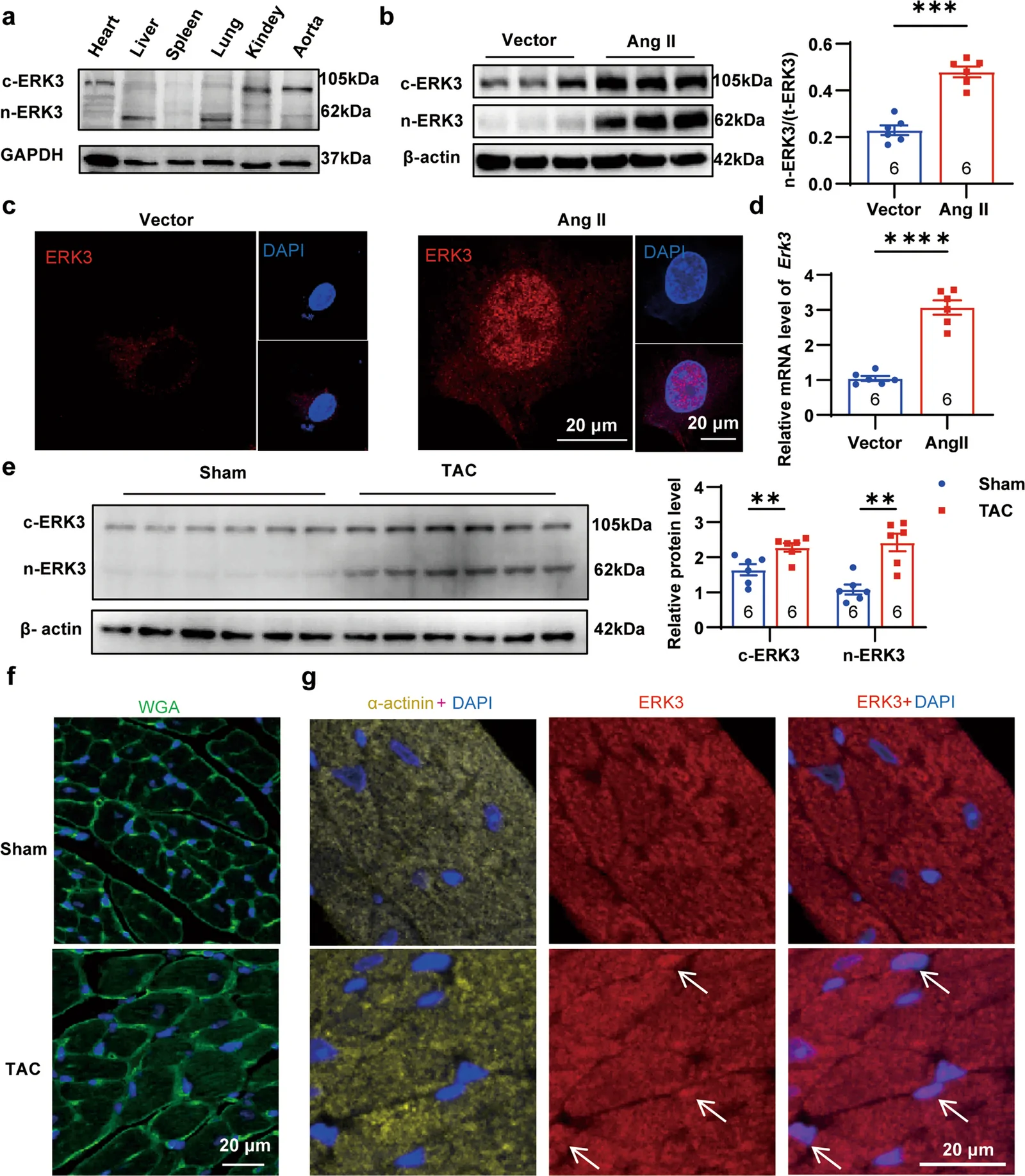
The increase of myocardial extracellular signal regulated kinase (ERK3) in hypertrophic cardiomyopathy is more significant in the nucleus. **a** Protein levels of intranuclear ERK3 (n-ERK3) and intracytoplasmic ERK3 (c-ERK3) in different mice tissues (heart, liver, spleen, lung, kidney and aorta). **b** Protein levels of n-ERK3 and c-ERK3 in neonatal rat primary cardiomyocytes (NRCMs) stimulated with or without Ang II for 24 hours (*n=*6). **c** Immunofluorescence (IF) staining of ERK3 in NRCMs stimulated with or without Ang II for 24 hours. Red: ERK3; Blue: 4’,6-diamidino-2-phenylindole (DAPI). Scale bar: 20 μm.** d** The mRNA levels of *Erk3* in NRCMs stimulated with or without Ang II for 24 hours (*n=*6). **e** Protein levels of n-ERK3 and c-ERK3 in the cardiomyocytes of mice after 4 weeks of transverse Aortic Constriction (TAC) or sham operation (*n=*6). **f** Wheat Germ Agglutinin (WGA) in heart tissues from mice after 4 weeks of TAC or sham operation. Scale bar: 20 μm. **g** IF staining of mice hearts after 4 weeks of TAC or sham operation. Red: ERK3; Yellow: alpha actinins (α-actin); Blue: DAPI. Scale bar: 20 μm. The data are presented as the mean ± standard error of the mean (SEM). A two-tailed Student’s t test was applied for **b, d** and** e**. **p<0.01, ***p<0.001, ****p<0.0001.

To the investigate the change of myocardial ERK3 in pathological cardiac remodeling evoked by pressure overload, we assessed the levels of ERK3 in vitro and in vivo. First, we measured the expression of ERK3 in different mouse tissues (heart, liver, spleen, lung, kidney and aorta) and observed that the form of ERK3 across different tissues (Fig. 1a). The full-length form of ERK3 has a molecular weight of approximately 105 kDa. Upon translocation into the nucleus, the C-terminal tail of ERK3 is cleaved, resulting in the formation of a truncated form of approximately 62 kDa. The 62 kDa endogenous ERK3 protein (labelled n-ERK3) was constitutively nuclear, whereas the 105 kDa endogenous ERK3 protein (labelled c-ERK3) was constitutively cytoplasmic. Specifically, under normal conditions, ERK3 is located mainly in the cytoplasm of the heart (Fig. 1a).

We then detected the levels and location of ERK3 in Ang II-treated neonatal rat cardiomyocytes (NRCMs) by western blotting (WB) and immunofluorescence (IF), revealing upregulated ERK3 expression in Ang II-treated NRCMs, particularly in the nucleus (Fig. 1b, c and Fig. S1a). Quantitative reverse transcriptase polymerase chain reaction (qRT-PCR) revealed a marked upregulation of ERK3 mRNA (Fig. 1d). In vivo, pressure-overload-induced pathological cardiac remodeling was induced via transverse aortic constriction (TAC). Analysis of isolated mouse cardiomyocytes revealed upregulated ERK3 expression following pressure overload (Fig. 1e and Fig. S1b), particularly its pronounced nuclear accumulation (Fig. 1e). Similarly, IF staining of frozen left ventricular (LV) sections from mice revealed increased levels of ERK3 in the nuclei of cardiomyocytes after TAC treatment (Fig. 1f, g and Fig. S1c).

Overall, ERK3 upregulation and nuclear enrichment may represent hallmarks of the hypertrophic response in both cell-based and whole-animal models.

### Cardiomyocyte-specific *Erk3* deficiency protects against pathological cardiac remodeling and cardiac dysfunction

**Fig. 2:**
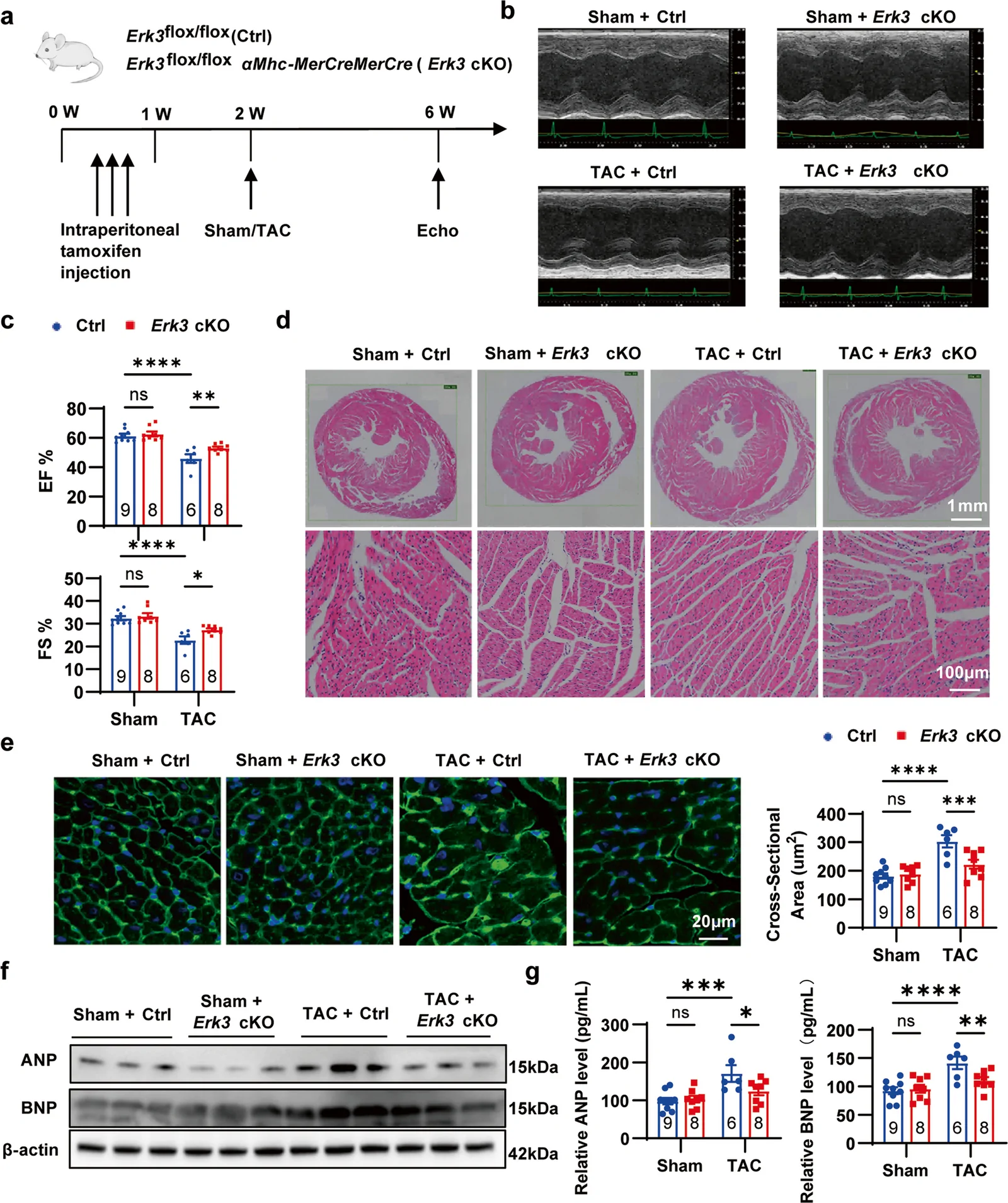
Cardiomyocyte-specific *Erk3* deficiency protects against pressure overload-induced pathological myocardial remodeling and cardiac dysfunction. **a** Schematic diagram of mouse animal modeling process. **b** Representative echocardiographic (Echo) image of *Erk3*^flox/flox^ (Ctrl) and *Erk3*^flox/flox^*αMhc-MerCreMer*^Cre^ (*Erk3* cKO) mice after 4 weeks of TAC or sham operation. **c** Ejection fraction (EF) and fractional shortening (FS) of Ctrl and *Erk3* cKO mice after 4 weeks of TAC or sham operation (*n* = 6 or 8 or 9). **d** Hematoxylin & eosin(H&E) staining of heart tissues from Ctrl and *Erk3* cKO mice after 4 weeks of TAC or sham operation. Scale bar: 1 mm or 100 μm. **e** Pooled cross-sectional area of cardiomyocytes in heart tissues from Ctrl and *Erk3* cKO mice after 4 weeks of TAC or sham operation (*n* = 6 or 8 or 9). Scale bar: 50 μm. **f** The protein levels of atrial natriuretic peptide (ANP) and B-type natriuretic peptide (BNP) in heart tissues from Ctrl and *Erk3* cKO mice after 4 weeks of TAC or sham operation.** g** The levels of ANP and BNP in the serum of Ctrl and *Erk3* cKO mice after 4 weeks of TAC or sham operation (*n* = 6 or 8 or 9). The data are presented as the mean ± SEM. One-way ANOVA with the Tukey multiple comparison test was applied for **c**, **e** and **g**. *p < 0.05, **p < 0.01, ***p < 0.001, ****p < 0.0001, ns = not significant

To assess the impact of myocardial ERK3 on pathological cardiac remodeling and contractile function in vivo, we generated cardiac-specific *Erk3* knockout mice (deletion confirmed in Fig. S2) and subjected them to TAC. Two weeks after tamoxifen administration, the mice underwent either TAC or a sham procedure (Fig. 2a). Echocardiographic evaluation revealed that TAC markedly impaired LV contractile function, as evidenced by significant decreases in the ejection fraction (EF) and fractional shortening (FS), which were notably reversed in *Erk3*^flox/flox^*αMhc-MerCreMer*^Cre^ (*Erk3* cKO) mice (Fig. 2b, c). Furthermore, following a four-week period of TAC, pathological cardiac remodeling and cardiac dysfunction were significantly alleviated in the mice, as evidenced by a reduction in heart size, LV wall thickness, and heart weight/tibia length (HW/TL) ratios in comparison to the those in the *Erk3* ^flox/flox^ (Ctrl) group (Fig. S3a). Deletion of *Erk3* in cardiomyocytes did not affect the survival rate of mice during the four-week observation period (Fig. S3b).

To determine the role of ERK3 in maladaptive remodeling, we assessed LV cardiomyocyte morphology. While TAC induced pronounced structural abnormalities in the Ctrl mice, these alterations were largely reversed in the *Erk3* cKO group (Fig. 2d). Four weeks after surgery, the increase in the myocyte cross-sectional area was significantly reduced in the *Erk3* cKO group (Fig. 2e). This antihypertrophic effect was further corroborated by the analysis of isolated cardiomyocytes, which exhibited a smaller cell size in TAC-treated *Erk3* cKO mice (Fig. S3c). Additionally, the expression of the molecular hypertrophic markers atrial natriuretic peptide (ANP) and B-type natriuretic peptide (BNP) increased response to pressure overload in the LV cardiomyocytes, a phenomenon that was significantly attenuated in *Erk3* cKO mice (Fig. 2f). Enzyme-linked immunosorbent assay (ELISA) test of mouse serum yielded similar results (Fig. 2g). We further analysed the expression of hypertrophic markers expression in Ang II-treated NRCMs. *Erk3* overexpression (verified in Fig. S4a-b) and knockdown (verified in Fig. S4c-d) increased and reduced the Ang II–induced upregulation of ANP and BNP respectively (Fig. S5).

**Fig. 3:**
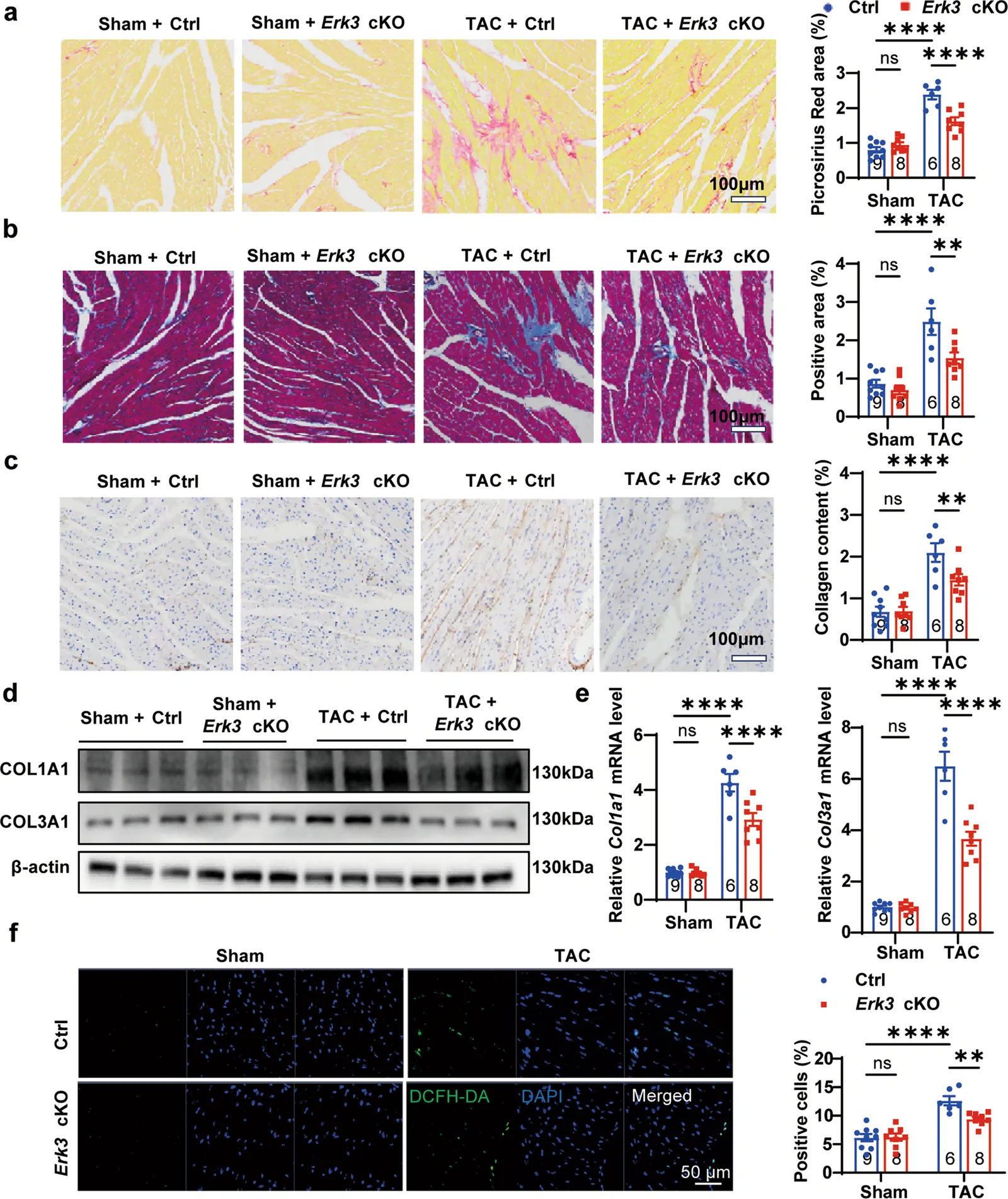
Cardiac-specific *Erk3* null ameliorate myocardial fibrosis induced by pressure overload.** a** Sirius Red stain in heart tissues from Ctrl and *Erk3* cKO mice after 4 weeks of TAC or sham operation (*n* = 6 or 8 or 9). Scale bar: 100 μm. **b** Masson staining in heart tissues from Ctrl and *Erk3* cKO mice after 4 weeks of TAC or sham operation (*n* = 6 or 8 or 9). Scale bar: 100 μm. **c** Collagen immunohistochemistry (IHC) dyeing in heart tissues from Ctrl and *Erk3* cKO mice after 4 weeks of TAC or sham operation (*n* = 6 or 8 or 9). Scale bar: 100 μm. **d** The protein levels of collagen type I alpha 1 chain (COL1A1) and collagen type I alpha 1 chain (COL3A1) in heart tissues from Ctrl and *Erk3* cKO mice after 4 weeks of TAC or sham operation. **e** The mRNA levels of *Col1a1* and *Col3a1* in heart tissues from Ctrl and *Erk3* cKO mice after 4 weeks of TAC or sham operation (*n* = 6 or 8 or 9). **f** 2’,7’-Dichlorodihydrofluorescein diacetate (DCFH-DA) in heart tissues from Ctrl and *Erk3* cKO mice after 4 weeks of TAC or sham operation (*n* = 6 or 8 or 9). The data are presented as the mean ± SEM. One-way ANOVA with the Tukey multiple comparison test was applied. **p < 0.01, ****p < 0.0001, ns = not significant

Given that ventricular myocardial fibrosis is the other characteristic feature of pathological cardiac remodeling, we next investigated the influence of ERK3 on collagen accumulation. Sirius-red and Masson’s trichrome staining demonstrated a reduction in cardiac fibrosis in *Erk3* cKO mice compared with that in Ctrl mice subjected to TAC (Fig. 3a, b). In *Erk3* cKO mice, collagen deposition was reduced in the heart under pressure overload, as indicated by the results of immunohistochemistry (IHC), WB and qRT-PCR (Fig. 3c-e). A reduction in reactive oxygen species (ROS) production was also noted in the *Erk3* cKO mice following TAC as demonstrated by 2’,7’-dichlorodihydrofluorescein diacetate (DCFH-DA) staining of cryopreserved cardiac tissue sections (Fig. 3f).

However, under basal conditions, cardiomyocyte-specific *Erk3* deletion had no discernible effect. This lack of a phenotype may be attributed to the minimal effect of *Erk3* knockdown on its nuclear pool under physiological conditions.

These findings collectively suggest that deleting *Erk3* in cardiomyocytes provides protection against TAC-induced pathological cardiac remodeling and dysfunction. This phenotypic neutrality likely stems from the fact that pathological nuclear accumulation of ERK3, rather than its mere presence, drives LV remodelling, a process that remains dormant under physiological conditions.

### Pressure overload may enhance ALY-ERK3 complex formation to mediate ERK3 nuclear transport via C-terminal tail removal

**Fig. 4:**
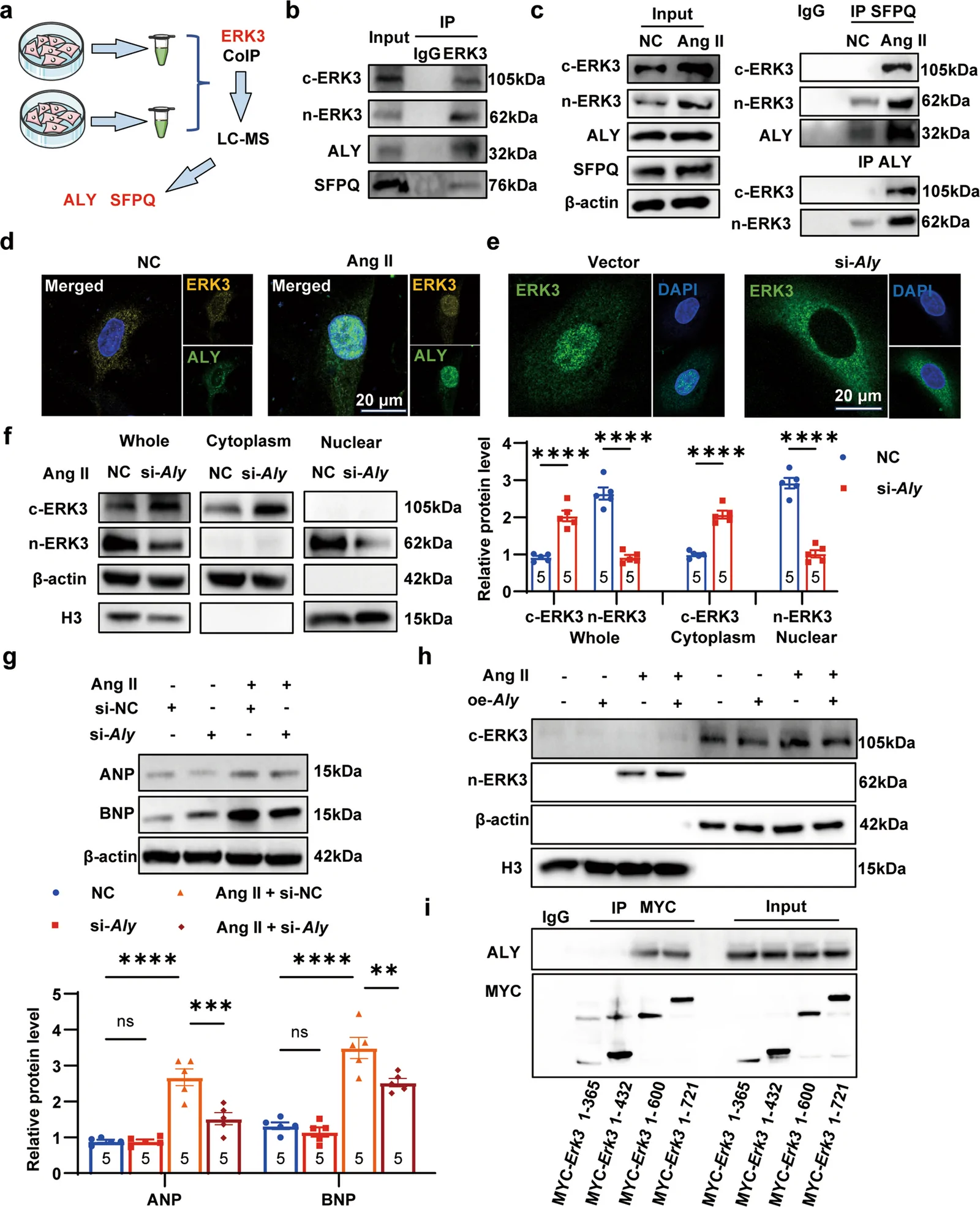
Aly/REF export factor (ALY) mediates the nuclear transportation of ERK3 via deleting the C-terminal tail of ERK3.** a** Diagram of the co-immunoprecipitation (CoIP) and liquid chromatography-tandem mass spectrometry (LC–MS/MS). **b** NRCMs were harvested and subjected to western bloting (WB) for n-ERK3, c-ERK3, ALY and splicing factor proline and glutamine rich (SFPQ) in the input and immunoprecipitates of IgG or anti-ERK3. **c** WB for n-ERK3, c-ERK3, SFPQ and ALY in the input and immunoprecipitates of IgG, anti-SFPQ or anti-ALY antibody in NRCMs treated with or without Ang II for 24 h. **d** IF for ERK3 and ALY in NRCMs treated with or without Ang II for 24 h. Yellow: ERK3; Green: ALY; Blue: DAPI. Scale bar: 20 μm. **e** IF for ERK3 in NRCMs transfected with the *Aly* siRNA (si-*Aly*) or control siRNA (si-NC) and treated with Ang II for 24 h. Green: ERK3; Blue: DAPI. Scale bar: 20 μm. **f** NRCMs transfected with the si-*Aly* or si-NC and treated with Ang II were harvested, and subjected to WB to access nucleoplasm distribution of n-ERK3 and c-ERK3 (*n* = 5). **g** The protein levels of ANP and BNP in NRCMs transfected with si-*Aly* or si-NC, with or without Ang II treatment for 24 h (*n* = 5). **h** NRCMs transfected with the *Aly* overexpression plasmids (oe-*Aly*) or control plasmids (NC) and treated with Ang II or not were harvested, and subjected to WB to access nucleoplasm distribution of n-ERK3 and c-ERK3. **i** NRCMs were transfected with Myc-*Erk3*_1–365Δ_, Myc-*Erk3*_1–432Δ_, Myc-*Erk3*_1–600Δ_ and Myc-*Erk3*_1–721_, and then CoIP was performed with an anti-MYC antibody, followed by ALY protein detection. The data are presented as the mean ± SEM. One-way ANOVA with the Tukey multiple comparison test was applied for **f** and **g**. **p < 0.01, ***p < 0.001, ****p < 0.0001, ns = not significant

While we have established that a deficiency in *Erk3* specifically in cardiomyocytes provides protection against cardiac dysfunction, the involvement of n-ERK3 or c-ERK3 remains unclear. To investigate the mechanism of ERK3 nuclear translocation, we performed CoIP followed by liquid chromatography-tandem mass spectrometry (LC–MS/MS) analysis and verified the results in cellular experiments. After screening the top 20 proteins (details in the Materials and methods section), we identified ALY and SFPQ as potential proteins that mediate the nuclear transport of ERK3 (Fig. 4a). Further experiments revealed that Ang II stimulation increased the binding of ALY and SFPQ to ERK3, especially n-ERK3 in the nucleus (Fig. 4b-d). Additionally, only *Aly* depletion significantly reduced Ang II–induced nuclear accumulation of ERK3 (Fig. 4e, f, the interference efficiency of *Aly* is verified in Fig. S6, and the negative result of SFPQ knockdown is not displayed). We then assessed hypertrophic marker expression in NRCMs transfected with the si-*Aly* or si-NC and treated with or without Ang II. The results indicated that the knockdown of *Aly* reversed the upregulation of ANP and BNP caused by Ang II stimulation (Fig. 4g). Similar results were obtained by WB, as evidenced by increased n-ERK3 and decreased c-ERK3 levels after the overexpression of *Aly* (Fig. 4h). The overexpression of *Aly* also increased the expression of hypertrophic markers in NRCMs under Ang II stimulation (Fig.S7a), and the knockdown of *Erk3* reversed the increase in ANP and BNP caused by *Aly* overexpression (Fig. S7b). Subsequent research, which included the development of ERK3 subclones with various amino acid fragments tagged with MYC, indicated that the essential binding domain for ALY is located within the amino acid sequence spanning residues 433 to 721 of ERK3 (Fig. 4i). This region is near the C-terminal tail of ERK3.

Nonetheless, neither the depletion nor the overexpression of *Aly* elicited a discernible phenotype under basal conditions, suggesting that the interaction between ALY and ERK3 in cardiomyocytes is negligible at steady state.

Therefore, we hypothesise that pressure overload enhances ALY-ERK3 complex formation to mediate ERK3 nuclear transport via C-terminal tail removal, and that targeting this pathway may have potential therapeutic effects on pathological cardiac remodeling.

### ES may relieve pressure overload-induced pathological cardiac remodeling and dysfunction through the ALY-ERK3 complex

**Fig. 5:**
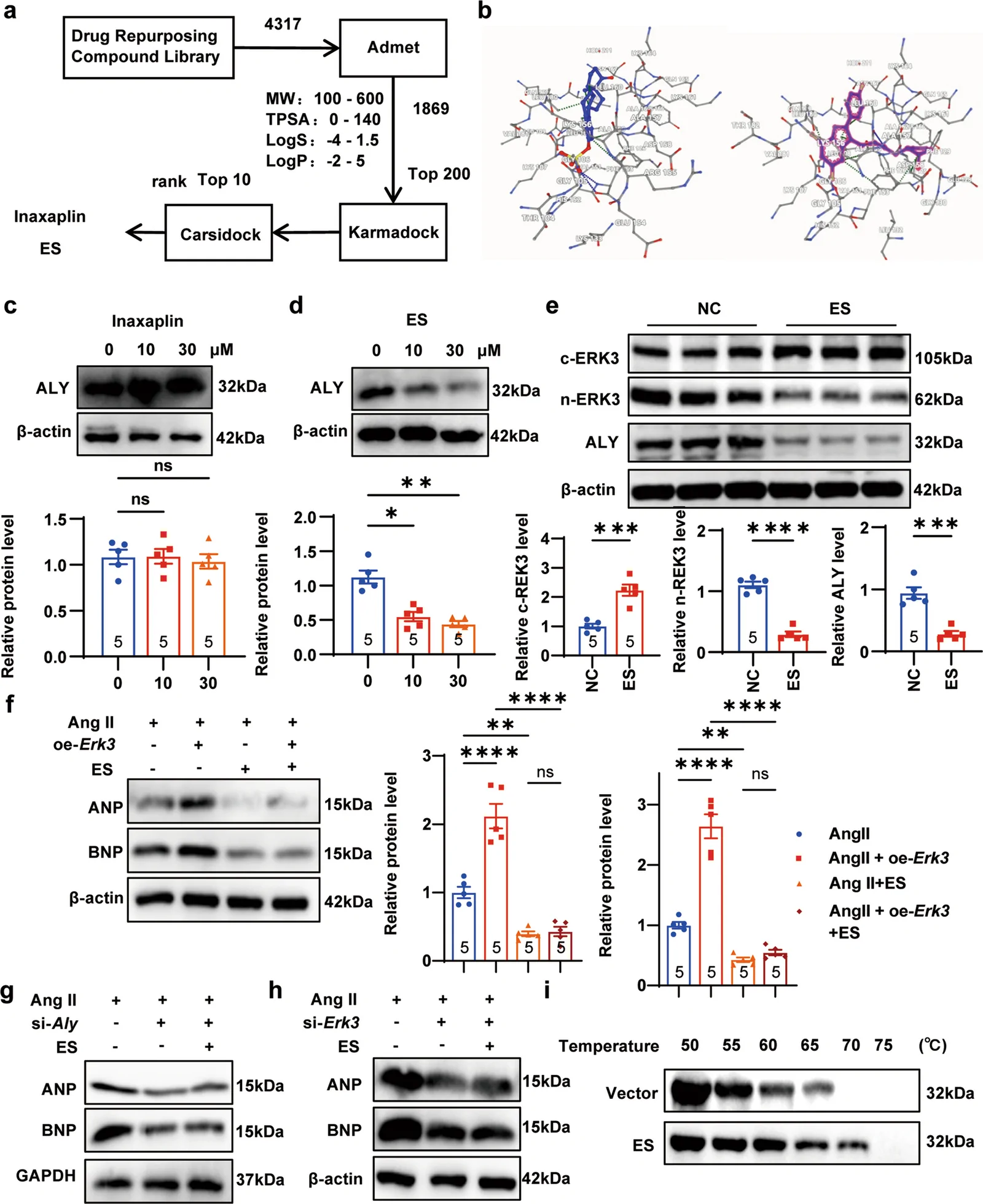
Estrone sulfate (ES) can reduce the level of ALY to promote cardiac dysfunction.** a** Schematic diagram of virtual Screening. **b** The two discovered targeted drug of ALY. **c-d** The protein levels of ALY in Ang II stimulated-NRCMs treated with ES and inaxaplin for 24 h (*n* = 5). **e** The protein levels of ALY, n-ERK3 and c-ERK3 in Ang II stimulated-NRCMs treated with or without ES for 24 h (*n* = 5). **f** The protein levels of ANP and BNP in Ang II stimulated-NRCMs transfected with the *Erk3* overexpression plasmids (oe-*Erk3*) or NC and treated with or without ES for 24 h (*n* = 5). **g** The protein levels of ANP and BNP in Ang II stimulated-NRCMs transfected with the si-*Aly* or si-NC and treated with or without ES for 24 h (*n* = 5). **h** The protein levels of ANP and BNP in Ang II stimulated-NRCMs transfected with the siRNA (si-*Erk3*) and treated with or without ES for 24 h (*n* = 5). **i** CETSA of the combination of ES and ALY. The data are presented as the mean ± SEM. One-way ANOVA with the Dunnett multiple comparison test was applied for **c-d**. One-way ANOVA with the Tukey multiple comparison test was applied for **f–h**. A two-tailed Student’s t test was applied for **e**. *p < 0.05, **p < 0.01, ***p < 0.001, ****p < 0.0001, ns = not significant

Given that n-ERK3, which is mediated by the ALY-ERK3 complex, plays a significant role in hypertrophic cardiomyopathy, we conducted a virtual screening of pharmacological agents targeting ALY to enhance the therapeutic approach for this condition and validated their therapeutic efficacy in vitro and in vivo. After virtual screening, which was conducted using the Drug Repurposing Compound Library and based on the specific selection criteria (Fig. 5a, details in the Materials and methods), we ultimately chose ES and inaxaplin for further validation (Fig. 5b). In vitro experiments revealed that ES can significantly reduce the level of ALY (Fig. 5c, d). Moreover, ES reduced the expression of n-ERK3 and increased the expression of c-ERK3 in NRCMs stimulated with Ang II (Fig. 5e). The protein levels of hypertrophic markers were markedly reduced by ES in Ang II-treated NRCMs, even when *Erk3* was overexpressed (Fig. 5f). To further validate the drug’s specificity, the hypertrophic markers of Ang II-treated NRCMs that were transfected with or without the si-*Aly* or si-NC and treated with ES or not were assessed, indicating that ES cannot further function when *Aly* is knocked out (Fig. 5g and Fig. S8a). Knocking down *Erk3* also yielded similar results (Fig. 5h and Fig. S8b), supporting the notion that ES acts via the ALY-ERK3 pathway. Through a cellular thermal shift assay (CETSA), we preliminarily demonstrated the binding of ES to ALY (Fig. 5i). ALY is an RNA-binding protein that functions through the RNA recognition motif domain. To exclude the RNA binding functions of ALY, Ang II-treated NRCMs were transfected with the △ALY (ALY without an RNA recognition motif) or control plasmids, and the results indicated that △ALY can still lead to the nuclear aggregation of ERK3 (Fig. S9).

**Fig. 6:**
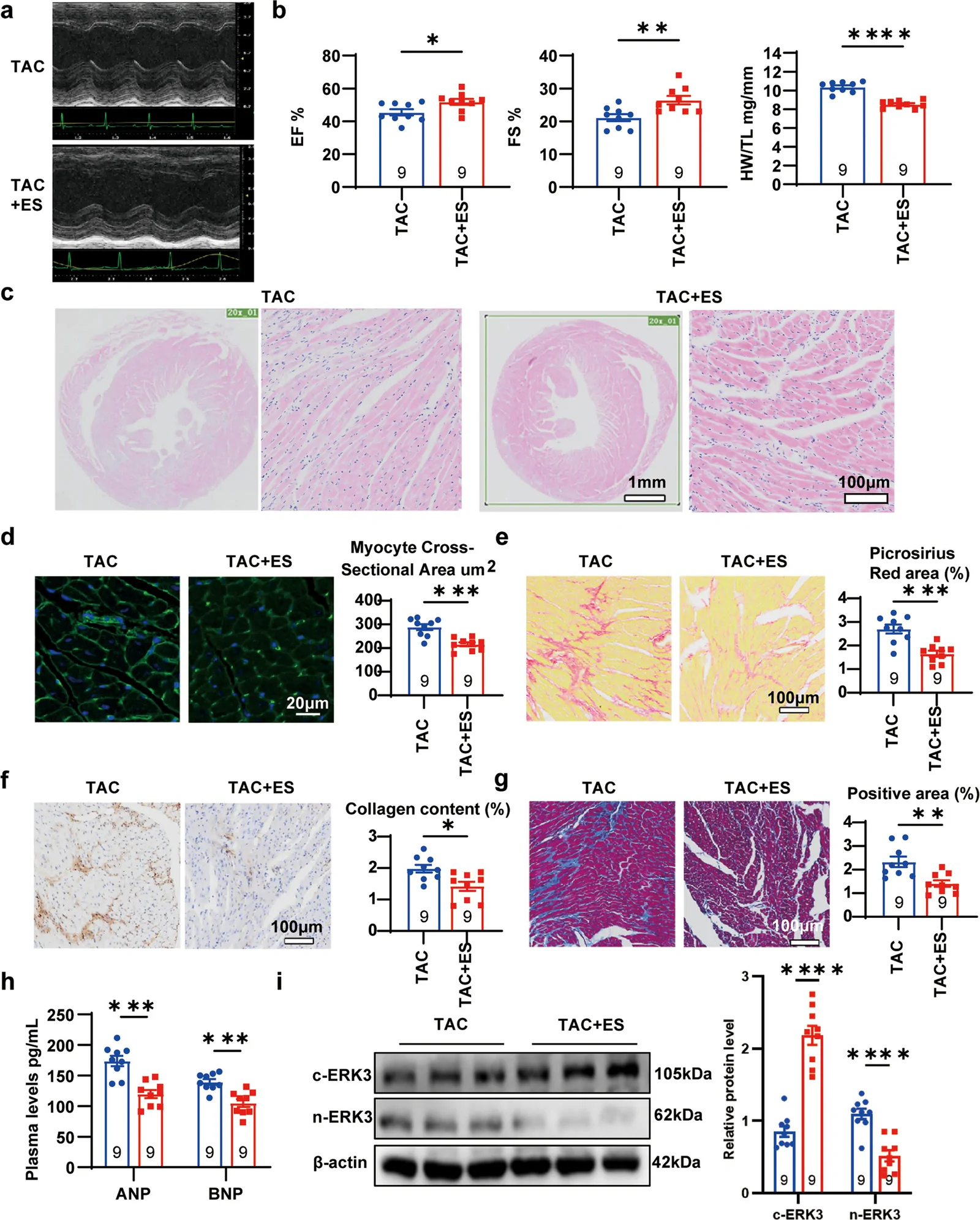
ES can relieve pathological myocardial remodeling through the ALY-ERK3 pathway. **a** Representative echocardiographic image of mice after 4 weeks of TAC operation treat with ES or not. **b** EF, FS and heart weight to tibia length ratio (HW/TL) of mice after 4 weeks of TAC operation treat with ES or not (*n* = 9). **c** H&E staining of heart tissues from mice treat with ES or not after 4 weeks of TAC operation. Scale bar: 1 mm or 100 μm. **d** Pooled cross-sectional area of cardiomyocytes in heart tissues from mice treat with ES or not after 4 weeks of TAC operation (*n* = 9). Scale bar: 20 μm. **e** Sirius Red** s**tain in heart tissues from mice treat with ES or not after 4 weeks of TAC operation (*n* = 9). Scale bar: 100 μm. **f** Collagen IHC dyeing in heart tissues from mice treat with ES or not after 4 weeks of TAC operation (*n* = 9). Scale bar: 100 μm. **g** Masson staining in heart tissues from mice treat with ES or not after 4 weeks of TAC operation (*n* = 9). Scale bar: 100 μm. **h** The levels of ANP and BNP in the serum of mice treat with ES or not after 4 weeks of TAC operation (*n* = 9). **i** Protein levels of n-ERK3 and c-ERK3 in heart tissues from mice treat with ES or not after 4 weeks of TAC operation (*n* = 9). The data are presented as the mean ± SEM. A two-tailed Student’s t test was applied for **b** and **d-i**. *p < 0.05, **p < 0.01, ***p < 0.001, ****p < 0.0001

To determine whether ES can pharmacologically target ALY and relieve contractile dysfunction in vivo, mice underwent TAC surgery and received ES or saline for four weeks. Echocardiographic assessment indicated that ES provides protection against contractile dysfunction of the heart (Fig. 6a, b and Fig. S10a). To further verify the therapeutic effect of ES on pathological myocardial remodeling, we conducted several histological staining experiments and showed that ES substantially reduced myocardial structural abnormalities and cardiac fibrosis in TAC model mice (Fig. 6c-g). ELISA of TAC mice serum revealed that the levels of the ANP and BNP decreased following treatment with ES (Fig. 6h). Furthermore, we measured ERK3 expression in the hearts of ES-treated mice. Similar to the results obtained in NRCMs, a decrease in ERK3 in the nucleus and an increase in ERK3 in the cytoplasm were observed (Fig. 6i). In addition, the hearts of ES-treated mice presented lower levels of hypertrophic markers and fibrotic markers (Fig. S10b).

Cumulative evidence suggests that ES has the potential to alleviate pressure overload-induced pathological cardiac remodeling and contractile dysfunction via the ALY-ERK3 complex. This may represent a promising therapeutic option for the treatment of pathological cardiac remodeling.

### The binding of the ALY-ERK3-SFPQ complex may hinder the inhibitory effect of SFPQ on *Txnip*

**Fig. 7:**
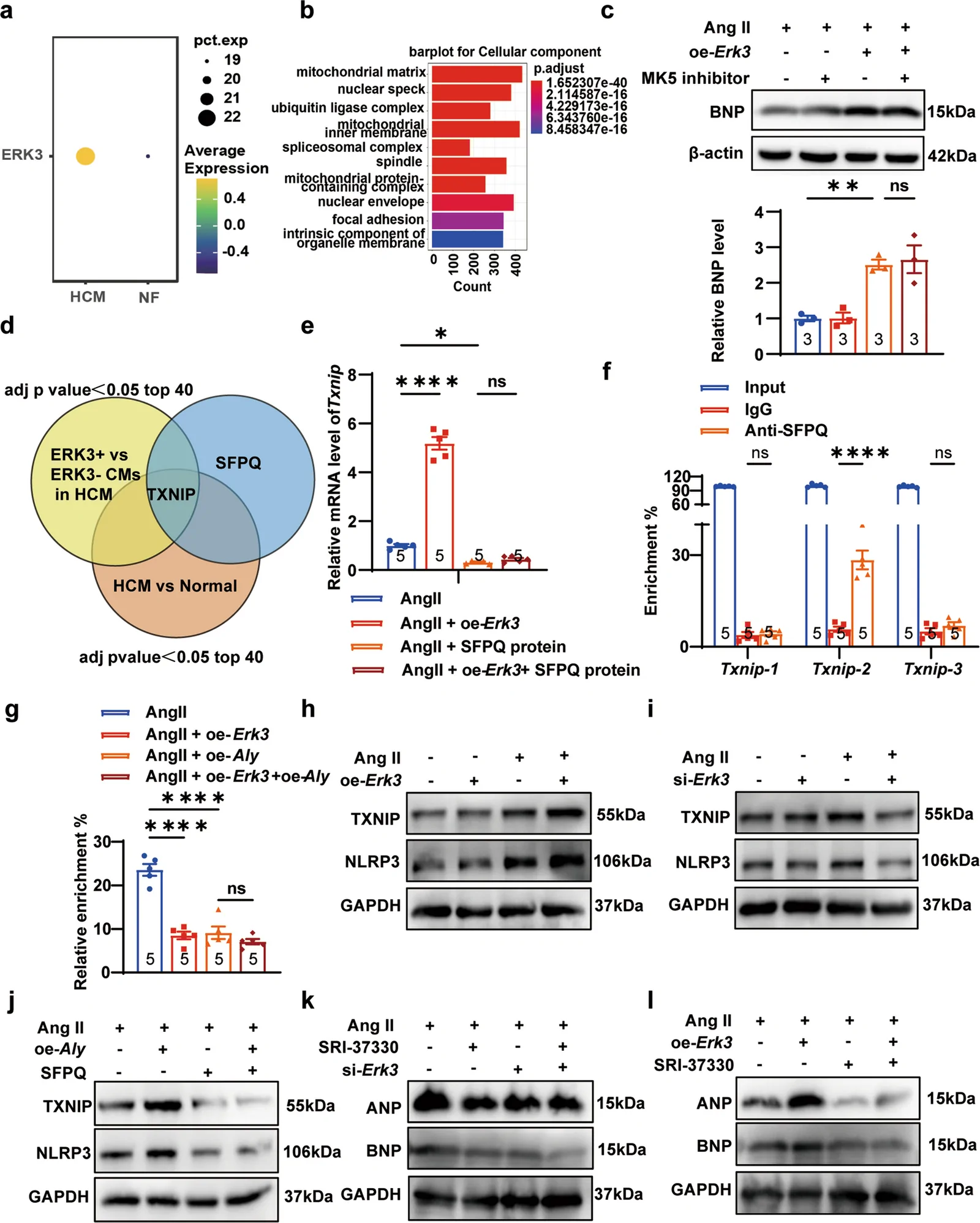
Nuclear transportation of ERK3 activates thioredoxin-interacting protein (TXNIP)/NOD-like receptor thermal protein domain associated protein 3 (NLRP3) pathway through SFPQ. **a** The levels of *ERK3* in single nucleus RNA sequencing (snRNA-seq) of hearts from hypertrophic cardiomyopathy (HCM) and no heart failure (NF) people. **b** Enrichment analysis of differentially expressed genes between myocardial cells with high and low expression of *ERK3* in HCM patients. **c** The protein levels of BNP in Ang II stimulated-NRCMs transfected with the oe-*Erk3* or NC and treated with or without mitogen activated protein kinase-activated protein kinase-5 (MK5) inhibitor GLPG0259 for 24 h (*n* = 3). **d** Venn Diagram. **e** The mRNA levels of *Txnip* in Ang II-treated NRCMs transfected with the oe-*Erk3* or NC and treated with or without SFPQ protein for 24 h (*n* = 5). **f** Validation of three *Txnip* primers for chromatin immunoprecipitation (ChIP) assay for SFPQ binding sites within *Txnip* promoter region (*n* = 5). **g** The relative interaction of *Txnip* and SFPQ in NRCMs tested by ChIP (% of input) for *Txnip*−2 fragment (*n* = 5). **h** The protein levels of TXNIP and NLRP3 in NRCMs transfected with the oe-*Erk3* or NC and treated with Ang II or not (*n* = 5). **i** The protein levels of TXNIP and NLRP3 in NRCMs transfected with the si-*Erk3* or si-NC and treated with or without Ang II for 24 h (*n* = 5). **j** The protein levels of TXNIP and NLRP3 in Ang II-treated NRCMs transfected with *Aly* overexpression plasmids (oe-*Aly*) or NC and treated with or without SFPQ protein for 24 h (*n* = 5). **k** The protein levels of ANP and BNP in NRCMs transfected with the si-*Erk3* or si-NC and treated with or without SRI-37330 for 24 h (*n* = 5). **l** The protein levels of ANP and BNP in Ang II stimulated-NRCMs transfected with the oe-*Erk3* and treated with or without SRI-37330 for 24 h (*n* = 5). The data are presented as the mean ± SEM. One-way ANOVA with the Tukey multiple comparison test was applied for **c**, **e** and **g**. A two-tailed Student’s t test was applied for **f**. *p < 0.05, **p < 0.01, ****p < 0.0001, ns = not significant

To validate the clinical relevance of our findings and explore the downstream mechanisms of ERK3, we analyzed single nucleus RNA sequencing (snRNA-seq) of LV samples from 15 patients with hypertrophic cardiomyopathy (HCM) and 16 nonfailing humans (NF) (Fig. S11) and verified the results. We found that the expression of ERK3 in cardiomyocytes from HCM was greater than that in NF, which was consistent with our results (Fig. 7a). To explore the mechanism by which ERK3 functions, we divided cardiomyocytes into those with high (*ERK3* +) and low *ERK3* expression (*ERK3*-). Enrichment analysis of differentially expressed genes in cardiomyocytes from the two groups of HCM hearts revealed that differentially expressed genes were closely related to mitochondrial related pathways (Fig. 7b). Moreover, MK5 inhibitors could not alleviate the increase in BNP induced by *Erk3* overexpression in Ang II-stimulated NRCMs (Fig. 7c), suggesting that ERK3 in cardiomyocytes exerts its effects through other mechanisms.

We have previously reported a specific increase in the SFPQ-ERK3 complex under Ang II stimulation, but SFPQ did not affect the nuclear entry of ERK3. SFPQ, an important DNA/RNA-binding protein that is enriched mainly in the nucleus, plays a central role in transcriptional regulation and other nuclear processes [19, 20]. Considering that ERK3 does not affect the expression level of SFPQ (Fig. S12), we hypothesised that the ERK3-SFPQ complex in the nucleus affects the downstream function of SFPQ. We then screened the genes that are affected by ERK3 in the cardiomyocytes of the hypertrophic cardiomyopathy group and compared them with genes whose expression differed between HCM and NF (Fig. 7d). *Txnip* emerged as a significantly altered SFPQ-related target (Fig. 7d), and its expression was confirmed to be modulated by *Erk3* under Ang II stimulation (Fig. 7e). SFPQ has been shown to bind to the *Txnip* promoter and inhibit the transcription of *Txnip* [21]. Chromatin immunoprecipitation (ChIP) indicated that the overexpression of *Erk3* or *Aly* can hinder the binding and inhibitory effects of SFPQ on *Txnip* (Fig. 7f, g), further confirming the activation effect of the ALY-ERK3-SFPQ complex on *Txnip*.

Taken together, the ALY-ERK3-SFPQ complex may hinder the inhibitory effect of SFPQ on *Txnip.*

### Nuclear transportation of ERK3 exacerbates hypertrophic cardiomyopathy through the TXNIP/NLRP3 pathway

**Fig. 8:**
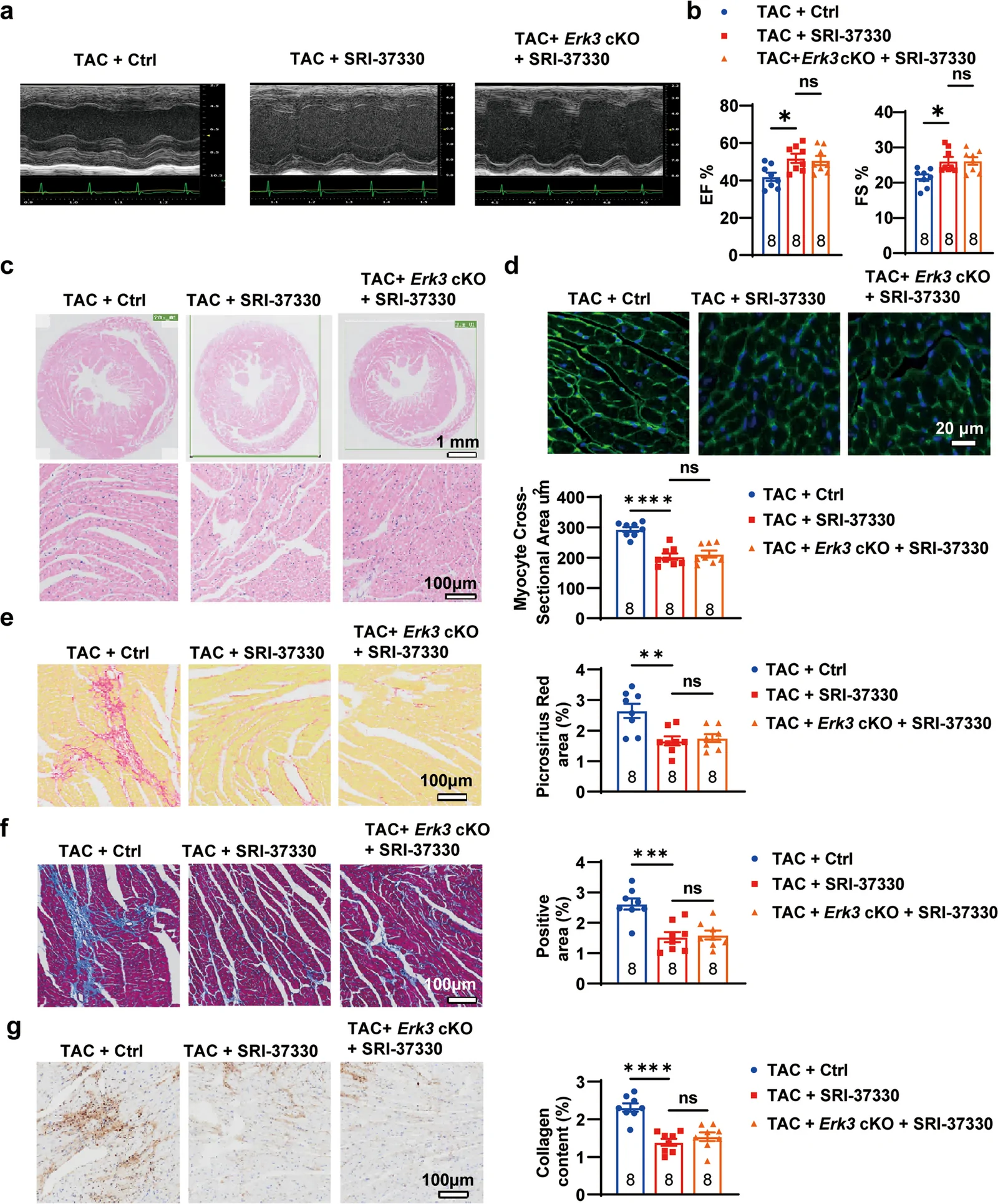
ERK3 exacerbates hypertrophic cardiomyopathy through TXNIP/NLRP3 pathway.** a** Representative echocardiographic image of Ctrl and *Erk3* cKO mice treat with SRI-37330 or not after 4 weeks of TAC operation. **b** EF and FS of Ctrl and *Erk3* cKO mice treat with SRI-37330 or not after 4 weeks of TAC operation (*n* = 8). **c** H&E staining of hearts from Ctrl and *Erk3* cKO mice treat with SRI-37330 or not after 4 weeks of TAC operation. Scale bar: 1 mm or 100 μm. **d** Pooled cross-sectional area of cardiomyocytes of hearts from Ctrl and *Erk3* cKO mice treat with SRI-37330 or not after 4 weeks of TAC operation (*n* = 8). Scale bar: 20 μm. **e** Sirius Red** s**tain of hearts from Ctrl and *Erk3* cKO mice treat with SRI-37330 or not after 4 weeks of TAC operation (*n* = 8). Scale bar: 100 μm. **f** Masson staining of hearts from Ctrl and *Erk3* cKO mice treat with SRI-37330 or not after 4 weeks of TAC operation (*n* = 8). Scale bar: 100 μm. **g** Collagen IHC dyeing of of hearts from Ctrl and *Erk3* cKO mice treat with SRI-37330 or not after 4 weeks of TAC operation (*n* = 8). Scale bar: 100 μm. The data are presented as the mean ± SEM. One-way ANOVA with the Tukey multiple comparison test was applied for **b** and **d-j**. *p < 0.05, **p < 0.01, ***p < 0.001, ****p < 0.0001, ns = not significant

Myocardial injury leads to oxidative stress and the release of ROS, which promote the interaction of TXNIP and NLRP3 to activate the NLRP3 inflammasome, thus increasing the expression levels of IL-1β and IL-18 and ultimately leading to pathological myocardial remodeling. However, whether ERK3 induces myocardial injury through the ROS-TXNIP-NLRP3 signaling pathway remains unclear. Through WB, we confirmed that the overexpression of *Erk3* under Ang II stimulation activated the TXNIP/NLRP3 pathway, whereas the downregulation of *Erk3* had the opposite effect (Fig. 7h, i and Fig. S13a-b). In addition, the SFPQ protein reversed the activation of TXNIP/NLRP3 caused by *Aly* overexpression, further demonstrating the effect of the ALY-ERK3-SFPQ complex on TXNIP (Fig. 7j and Fig. S13c). To further test this, we used SRI-3730, an orally bioavailable TXNIP inhibitor. SRI-3730 inhibited the expression of Ang II-mediated fibrotic and hypertrophic markers, but the loss of *Erk3* did not further decrease the expression of hypertrophic markers, suggesting that SRI-3730 and ERK3 target the same biological process of Ang II-induced hypertrophy (Fig. 7k and Fig. S13d). Similarly, *Erk3* overexpression did not exacerbate injury in the presence of SRI-3730 (Fig. 7l and Fig. S13e). In vivo, SRI-3730 improved contractile function in TAC-operated mice but did not confer any additional benefit when *Erk3* was specifically deleted in cardiomyocytes (Fig. 8a, b and Fig. S14a). Histological stainings showed similar conclusions (Fig. 8c-g). The NLRP3 inflammasome can induce the maturation and release of the proinflammatory cytokines IL-1β and IL-18 by activating caspase-1. We further revealed that SRI-3730 alleviated NLRP3 inflammasome activation (Fig. S14b), caspase-1 activity (Fig. S14c), and IL-1β expression (Fig. S14d) in the myocardial tissue of TAC mice, but no further improvement occurred after the specific knockout of *Erk3* in cardiomyocytes.

Collectively, these results indicate that ERK3 may induce oxidative stress and upregulate TXNIP in the mouse myocardium, thereby driving myocardial injury by regulating the ROS-TXNIP-NLRP3 signaling pathway.

## Discussion

In our study, we elucidated the role of myocardial ERK3 in modulating pressure stress-induced pathological cardiac remodeling. Mechanistically, the accumulation of myocardial ERK3 in the nucleus induced by pressure overload may exacerbate pathological cardiac remodeling through the SFPQ/TXNIP/NLRP3 pathway. ALY may mediate the transport of ERK3 by truncating the C-terminal tail of ERK3 in cardiomyocytes, and a newly reported ALY-targeted inhibitor, ES, has the potential to relieve pathological cardiac remodeling.

To date, only a few studies have linked ERK3 to heart disease. Previous research has consistently indicated that fibroblast-derived ERK3 is capable of binding to MK5 to reduce pathological myocardial remodeling and sustain contractile dysfunction, whereas cardiomyocyte-derived ERK3 lacks this interaction [9–12]. Our findings indicate that the downregulation of *Erk3* in cardiomyocytes can ameliorate pathological myocardial remodeling resulting from pressure stress.

ERK3 is translocated to the nucleus after its C-terminal tail is removed, and both the nuclear and cytoplasmic forms of ERK3 can be detected simultaneously using N-terminal antibodies [16]. Our research revealed that ERK3 expression varies across different normal tissues, and the protein is predominantly localised in the cytoplasm of normal myocardial tissue. Notably, under conditions of stress loading, there is a significant increase in nuclear ERK3 expression. Subsequent analyses revealed that although there is a modest increase in extracellular ERK3 levels, the function of myocardial ERK3 in stress-induced hypertrophic cardiomyopathy is primarily attributable to its intracellular activity.

The specific mechanism governing the entry of ERK3 into the nucleus has yet to be elucidated [22, 23]. Our study revealed a novel co-factor, ALY, that may facilitate ERK3 nuclear entry. ALY is a pivotal nuclear RNA-binding protein that plays a significant role in mRNA processing, transport, and quality control [24, 25]. ALY has been proven to binds to the N-myc proto-oncogene protein (MYCN) in the nucleus and modulates MYCN protein stability [26], however, studies on whether the binding of ALY to proteins regulates protein cytoplasmic and nuclear localisation are lacking. This study investigated its role in regulating protein nuclear shuttling, which has rarely been studied. At present, research on the role of ALY in cardiovascular diseases is limited. Fibroblastic ALY has been shown to regulate cardiac remodeling after myocardial infarction as a 5-methylcytidine reader, but the specific mechanism remains unclear [27, 28]. This study represents the first report on the involvement of ALY in cardiomyocytes, however, further research is warranted to elucidate the specific mechanisms involved.

Under physiological conditions, modulating ERK3 levels in cardiomyocytes has negligible effects on cardiac function. This observation parallels findings regarding ERK3 in fibroblasts from other studies [12]. Furthermore, *Aly* knockdown typically does not affect cardiac function, reinforcing the notion that ERK3 is infrequently expressed in the nucleus and that ALY binding to ERK3 is reduced under physiological conditions. Our previous research has indicated that ERK3 in endothelial cells is degraded in response to shear stress, a process that may facilitate the progression of atherosclerosis [29]. Given the differential roles of ERK3 across various diseases, the precise targeting of ERK3 within the nuclei of cardiomyocytes may represent a more effective therapeutic approach for pressure overload-induced myocardial injury.

Our findings reveal that ES can effectively target ALY, and the results of subsequent experiments indicate that ES has the potential to ameliorate stress-induced cardiac injury. ES is a bioactive estrogen and a principal circulating plasma estrogen, which is converted to the its active form estrone via steroid sulfate esterase. Although direct research linking ES to cardiomyopathy is currently lacking, numerous studies have explored the relationship between estrogen and cardiovascular health [30]. Notably, compared with that in age-matched men, the lower incidence of heart failure in postmenopausal women suggesting a cardioprotective role for estrogen [31]. Additionally, G protein-coupled estrogen receptors are implicated in the pathophysiology of stress-induced heart disease [32]. However, investigations into the effects of estrogen replacement therapy on cardiovascular health often yield conflicting results [33–35]. Our research indicates that ES can enhance outcomes in individuals with stress-induced cardiomyopathy but has no effect under normal physiological conditions. Consequently, the variability in the efficacy of estrogen therapy may be linked to the diverse underlying causes of cardiovascular damage. Moreover, leveraging specific estrogen analogues could offer enhanced clinical benefits.

In addition, through analyzing snRNA-seq of LV samples from 15 hypertrophic cardiomyopathy patients and 16 nonfailing humans [36], we found that, unlike ERK3 in fibroblasts, ERK3 in cardiomyocytes promoted myocardial fibrosis and cardiac function injury by activating the TXNIP/NLRP3 pathway after nuclear entry. Specifically, the ALY-ERK3 complex can bind to SFPQ, hindering the binding and inhibitory effects of SFPQ on TXNIP, and thus activating the TXNIP/NLRP3 pathway. TXNIP is an oxidative stress-related gene that leads to ROS release and promotes the interaction of TXNIP and NLRP3 to activate the NLRP3 inflammasome, thus increasing the expression levels of IL-1β and IL-18 and ultimately leading to pathological cardiac remodeling [37–40]. This is the first study to report the ALY-ERK3-SFPQ complex and elucidate the relationships between ERK3 and TXNIP or NLRP3.

Our research has several limitations. First, although we confirmed that the ALY fragment binds to ERK3, there are no ready-made modelling data for ERK3 in this fragment, making accurate simulation of the binding site difficult. Future studies should aim to accurately target the binding sites of ERK3 and ALY. Second, when specific downstream mechanisms are being studied, accurate control of the protein content of ERK3 in the nucleus is difficult. A more precise approach will be necessary in future investigations. Furthermore, the existence and function of the ALY-ERK3-SFPQ complex require further experimental verification. Given that ALY is a well-characterized RNA-binding protein and nuclear export factor, and that the biological function of the interaction between ALY and full-length ERK3 remains insufficiently characterized, whether ALY directly regulates ERK3 C-terminal truncation remains to be further validated.

In conclusion, our study elucidates the protective role of downregulating myocardial cell ERK3 against stress-induced cardiac loads and investigates the potential mechanism through which ERK3 is activated under such conditions. This research broadens the understanding of the involvement of ERK3 in heart disease and contributes to the understanding of ERK3 nuclear transport mechanisms. In summary, targeting the ALY-ERK3-SFPQ complex may represent a promising therapeutic strategy for the treatment of pathological cardiac remodeling and heart failure.

## Materials and methods

### Animals

Genetically modified mice were developed by Cyagen (Suzhou) Biotechnology Co., Ltd. *Erk3* cKO mice were produced by crossing *Erk3*^flox/flox^ mice with *αMhc-MerCreMer*^Cre^ transgenic mice. Target gene manipulation was achieved through intraperitoneal administration of tamoxifen (MCE, HY-13757) at a dosage of 10 mg/kg for a duration of 7 days. Ctrl mice received the same tamoxifen dosage. ES (20 mg/kg/d) or SRI-37330 (100 mg/kg/d) were intragastric administrated. All mice were maintained with adequate sterilized water and food, adhering to a 12-h light/dark cycle.

### Cell extraction and culture

Hearts were surgically excised from 1 day-old neonatal rats, minced, and subjected to sequential enzymatic digestion. The dissociated cell suspension was filtered through a 70 µm mesh to remove tissue debris, followed by differential plating for 4 h to reduce fibroblast contamination. 0.1 mM 5-bromo-2’-deoxyuridine (BrdU) was added to the culture medium. Spontaneous contractions were typically observed within 24–72 h, confirming functional cardiomyocyte viability. Purity of the cardiomyocyte was determined by IF. Pressure overload was induced by treated with 1 μm Ang II (sigma, A9525) for 24 h. Cells were treated with ES (10 nm) or SRI-37330 (1um) for 24 h.

Adult mice were heparinized and anesthetized, followed by rapid heart excision. Hearts were rapidly cannulated via the aorta and perfused with Ca^2+^-free Tyrode’s solution at 37 °C for 5 min, followed by digestion with collagenase type II for 15–20 min. The digested ventricles were minced and gently triturated to release individual cells. The suspension was filtered and centrifuged, and Ca^2+^concentration was gradually restored to 1 mM by stepwise additions. Rod-shaped cells with clear striations were considered viable. Cardiomyocyte identity was confirmed by IF.

### Transverse aortic constriction (TAC)

Two weeks post-tamoxifen administration, the mice underwent either TAC or sham surgery. 8-week-old mice were anesthetized using isoflurane. An incision was made in the left thoracic region, and a 7.0 nylon suture ligature was secured around a 27-gauge needle positioned at the transverse aorta to achieve a 65–70% constriction upon needle removal. The sham operation was executed using an analogous technique, but without the application of ligation.

### Western blotting (WB)

Tissue or cell lysates were prepared using radio immunoprecipitation assay buffer (RIPA) containing protease and phosphatase inhibitors, and protein concentrations were quantified via bicinchoninic acid assay (BCA). Equal amounts of protein were separated by sodium dodecyl sulfate–polyacrylamide gel electrophoresis (SDS-PAGE) and transferred onto polyvinylidene fluoride (PVDF) membranes. After blocking with 5% non-fat milk in tris-buffered saline with tween-20 (TBST) for 1 h, membranes were incubated overnight at 4 °C with primary antibodies, followed by horseradish Peroxidase (HRP)-conjugated secondary antibodies for 1 h at room temperature. Protein bands were visualized using enhanced chemiluminescence reagents and imaged with a ChemiDoc system (Bio-Rad). Band intensities were quantified using Image J. All antibodies used for WB are shown in Table S1.

### IF staining

Cells are seeded on coverslips, fixed with paraformaldehyde, and permeabilized with a detergent like Triton X-100 to allow antibody access. Tissue slices undergo deparaffinization and antigen retrieval if needed. Both samples are blocked with bovine serum albumin (BSA) to prevent non-specific binding. Primary antibodies targeting the protein of interest are applied and incubated, followed by washing to remove unbound antibodies. Fluorescently labeled secondary antibodies are then added to bind the primary antibodies. Nuclei are counterstained with 4’,6-diamidino-2-phenylindole (DAPI). After final washes, coverslips or slides are mounted with an antifade medium and imaged using fluorescence confocal microscope. All antibodies used included in Table S1.

### RNA extraction and qRT-PCR analysis

Total RNA is extracted from cells or tissues using TRIzol reagent, followed by DNase treatment to remove genomic DNA contamination. RNA concentration and purity are assessed using a spectrophotometer or bioanalyzer. For qRT-PCR, RNA is reverse-transcribed into complementary DNA (cDNA) and then used as a template for qPCR, where gene-specific primers and SYBR Green are employed to amplify and quantify target sequences in real-time. Data are analyzed using the comparative Ct (ΔΔCt) method, normalizing target gene expression to housekeeping genes and calculating fold changes relative to a control group. All primers are shown in Table S2.

### Mouse tail genotyping

Genomic DNA was extracted from mouse tail biopsies for genotype identification. Mouse tail genotyping was carried using Quick Genotyping Assay Kit for Mouse Tail (Beyotime) according to the manufacturer’s protocol. PCR products were resolved by 2% agarose gel electrophoresis, stained with GelRed, and visualized under UV light. Primers used were shown in Table S2.

### Cell transfection

siRNA transfection was conducted when the cells reached approximately 60%−70% confluence. The specific siRNA sequences utilized are detailed in Table S3. Plasmid transfection was carried out when the cells attained approximately 90%−100% confluence. The transfection was facilitated using Lipofectamine™ 3000 Transfection Reagent, in accordance with the manufacturer’s guidelines.

### Cardiac functions examination

Mice are anesthetized and placed in a supine position on a warming pad to maintain body temperature. The chest is shaved, and ultrasound gel is applied to ensure proper acoustic coupling. A high-frequency transducer (30–40 MHz) is used to obtain two-dimensional (2D) and M-mode images of the heart in parasternal long-axis and short-axis views at the level of the papillary muscles.

### Hematoxylin & eosin (H&E)

Tissue sections are stained with hematoxylin, a basic dye that binds to acidic structures. After rinsing in water, sections are differentiated in a weak acid solution to remove excess stain and then blued in a weak alkaline solution to enhance nuclear staining. Next, sections are counterstained with eosin, an acidic dye that binds to basic structures. After dehydration through graded ethanol and clearing in xylene, sections are mounted with a coverslip using a permanent mounting medium.

### Immunohistochemistry (IHC)

Antigen retrieval is then performed using heat-induced epitope retrieval (HIER) or enzymatic digestion to unmask epitopes. Endogenous peroxidase activity is blocked with hydrogen peroxide, and non-specific binding is minimized by incubating sections with a blocking serum. Primary antibodies specific to the target protein are applied and incubated, followed by washing to remove unbound antibodies. A secondary antibody conjugated to an horseradish peroxidase is then applied. The signal is visualized using diaminobenzidine, which produces a colored precipitate at the site of the target protein. Sections are counterstained with hematoxylin to highlight nuclei, dehydrated, cleared in xylene, and mounted with a coverslip.

### Cardiomyocyte cross-sectional area detection

Sections are deparaffinized, rehydrated, and stained using Wheat Germ Agglutinin (WGA) conjugated to a fluorescent dye to outline cell membranes. Sections are incubated with the fluorescent conjugate, washed, and mounted with an antifade medium containing DAPI to label nuclei. Images are captured using fluorescence confocal microscope. Using ImageJ, the cross-sectional area of individual cardiomyocytes is traced and quantified based on membrane or boundary staining.

### Masson’s trichrome staining

Sections are stained with Weigert’s iron hematoxylin to highlight nuclei. After rinsing, sections are treated with Biebrich scarlet-acid fuchsin solution. Next, sections are differentiated in phosphomolybdic–phosphotungstic acid solution to remove excess red stain from collagen. The sections are then stained with aniline blue or light green. After rinsing, sections are dehydrated through graded ethanol, cleared in xylene, and mounted with a coverslip using a permanent mounting medium.

### Sirius-red staining

Sections are stained with Weigert’s hematoxylin to highlight nuclei. After rinsing, sections are incubated in a saturated solution of Picro-Sirius Red for 1 h. The sections are then rinsed in acidified water to remove unbound dye and differentiate the staining. After dehydration through graded ethanol and clearing in xylene, sections are mounted with a coverslip using a permanent mounting medium.

### 2’,7’-dichlorodihydrofluorescein diacetate (DCFH-DA)

Tissue slices are incubated with DCFH-DA in PBS for 30–60 min at 37 °C. After washing with buffer to remove excess probe, slices are treated with experimental stimuli to induce ROS production. Slices are then rinsed, mounted on slides, and imaged using a fluorescence microscope. Fluorescence intensity, proportional to ROS levels, is quantified using image analysis software.

### Enzyme-linked immunosorbent assay (ELISA)

Serum concentrations of biomarkers, specifically ANP and BNP, were measured in murine subjects utilizing commercially available assay kits designed for ANP and BNP, respectively (E-EL-M0166, E-EL-M0204, Elabscience). Protein samples isolated from murine hearts were tested for IL-1β applying a mouse ELISA kit (MLB00C; R&D Systems) according to the manufacturer’s recommendations.

### Caspase-1 activity

Protein samples isolated from murine hearts were treated with a selective caspase-1 inhibitor, and measured for caspase-1 activity applying Caspase-Glo 1 Inflammasome Assay (Promega, Madison, Wisconsin, USA). Luminescence was measured by using a plate-reading luminometer (GloMax-Multi Detection System; Promega). A mouse recombinant active caspase-1 (1181–100, lot 6C11L11810; BioVision, Milpitas, CA, USA) was used for a calibration curve.

### Co-immunoprecipitation (CoIP)

Cells were subjected to treatment with a lysis buffer, protease inhibitor cocktails, and NP-40. Following centrifugation, the supernatant fractions underwent IP utilizing monoclonal IgG along with the requisite antibodies, which were incubated on protein-A/G beads. The resulting precipitates were subsequently analyzed through SDS-PAGE followed by immunoblotting.

### Chromatin immunoprecipitation (ChIP)

ChIP DNA-end was repaired to overhang a 3’-dA, then adapters were ligated to the end DNA fragments. DNA fragments with proper size were selected after PCR amplification.

### LC‒MS/MS

Cell lysates underwent immunoprecipitation utilizing anti-ERK3 magnetic beads. The resulting precipitates were subjected to separation via SDS-PAGE and subsequently stained with Coomassie blue. The stained protein bands were excised into smaller fragments and digested to facilitate peptide extraction. For proteomic data analysis, liquid chromatography-tandem mass spectrometry was employed to analyze the extracted peptides (GeneSky Biotechnologies Co.Ltd.). Two independent experiments was conducted and the relative increasing ratio of the proteins binding to ERK3 under AngII stimulation was calculated. After removing the RP protein, simple membrane proteins and simple nuclear proteins, we selected the top 50 proteins with the highest changes for intersection, and finally obtained 20 proteins (Table S4). Through further CoIP verification, we determined that SFPQ and ALY can bind to ERK3 and increase their binding under the stimulation of AngII.

### Virtual screening

Virtual screening was conducted by DrugFlow. Drug Repurposing Compound Library which collects 4,317 approved and clinically entered drugs was sellected for virtual screening. 3ulh were downloaded from Protein Data Bank as the protein structure of ALYREF. To exclude compounds with poor physical and chemical parameters to reduce screening costs, we set the threshold: MW 100—600, TPSA 0—140, LogS −4—1.5, LogP −2—5, and retain 1869 compounds with certain drug likeness characteristics. Top 200 drugs selected by karmadock were then put into carsidock. Based on carsiscore and rtmscore, we finally chose ES and inaxaplin for further validation.

### snRNA-seq analysis

snRNA-seq of LV samples from 15 hypertrophic cardiomyopathy and 16 non-failing human were download from Single Cell Portal (SCP1303) and loaded into R v4.3.0. Clustering results obtained via unsupervised graph-based clustering partitioning were visualized by UMAP embedding. Differentially expressed genes were identified using a threshold of padj < 0.05 and a |log_2_FC|> 1.

### Cellular thermal shift assay (CETSA)

Cells were harvested, resuspended in PBS, and aliquoted into PCR tubes. The cell suspensions were subjected to a temperature gradient for 5 min using a thermal cycler, then cooled to room temperature. Cells were lysed and centrifuged at 20,000 × g for 20 min at 4 °C to separate soluble proteins from precipitated proteins. The supernatants containing soluble proteins were collected, and the amount of ALY remaining in the soluble fraction was quantified by WB.

### Statistics

All data are presented as mean ± standard error of the mean (SEM). The analysis was conducted utilizing GraphPad Prism 9 software. A *p*-value of less than 0.05 was deemed statistically significant.

## Supplementary Information

Supplementary Material 1.

## Data Availability

Raw metabolomics sequencing are provided in Figshare (https://doi.org/10.6084/m9.figshare.32968700). Source data are provided with this paper. Raw data will be available upon request.
