# Supplementary material for: Extracellular signal-regulated kinase 3 forms a nuclear complex with Aly/REF export factor and the splicing factor proline and glutamine rich to exacerbate pathological cardiac remodeling due to pressure overload
Source: Mol Biomed. 2026 Sep 18;7:177. doi: 10.1186/s43556-026-00563-9 (PMC13586137; doi:10.1186/s43556-026-00563-9)
Supplement: Supplementary file 1 [file 43556_2026_563_MOESM1_ESM.docx]

**SUPPLEMENTAL MATERIAL**

**Extracellular signal-regulated kinase 3 forms a nuclear complex with Aly/REF export factor and the splicing factor proline and glutamine rich to exacerbate pathological cardiac remodeling due to pressure overload**

Wen-Ying Zhou^1 †^, Feng Wang^1 †^, Shu-Yu Wang^1 †^, Li-Guo Wang^2 †^, Ai-Qun Chen^1^, Haoyue Tang^1^, Ya-Peng Chen^1^, Huan Zhang^1^, Xiao-Fei Gao^1^, Juan Zhang^1^*, Shao-Liang Chen^1^*, Jun-Jie Zhang^1^*

^1^Division of Cardiology, Nanjing First Hospital, Nanjing Medical University, 68 Changle Road, Nanjing, China.

^2^Key Laboratory of Cardiovascular Intervention and Regenerative Medicine of Zhejiang Province, Department of Cardiology, Sir Run-Run Shaw Hospital, School of Medicine, Zhejiang University, Hangzhou, China

Wen-Ying Zhou, [mkwenying929@163.com;](mailto:mkwenying929@163.com;)

Feng Wang, doctorfengwang@163.com;

Shu-Yu Wang, wangshuyu2332@163.com;

Li-Guo Wang, berniew1121@163.com;

Ai-Qun Chen, yeqicaq@163.com;

Haoyue Tang, [jsdfthy@163.com;](mailto:jsdfthy@163.com;)

Ya-Peng Chen, njmuchenyapeng@126.com;

Huan Zhang, [zhang07182022@163.com;](mailto:zhang07182022@163.com;)

Xiao-Fei Gao, [gaoxfcy@163.com](mailto:gaoxiaofei2014@163.com);

*Corresponding authors: Jun-Jie Zhang, [jameszll@163.com](mailto:jameszll@163.com); jameszll@njmu.edu.cn;

Shao-Liang Chen, [chmengx@njmu.edu.cn;](mailto:chmengx@njmu.edu.cn;) [chmengx@126.com](mailto:chmengx@126.com);

Juan Zhang, yinyinye9157793@126.com;

† These authors contributed equally to this work.

**Figure S1. The levels of myocardial extracellular signal regulated kinase (ERK3) increase under pressure overload. a** The mRNA levels of B-type natriuretic peptide **(***Bnp*) in neonatal rat primary cardiomyocytes (NRCMs) stimulated with or without Ang Ⅱ for 24 hours (n=6). **b** The mRNA levels of *Erk3* in the cardiomyocytes of mice after 4 weeks of transverse aortic constriction (TAC) or sham operation (n=6). **c** Pooled cross-sectional area of cardiomyocytes in heart tissues from mice after 4 weeks of TAC or sham operation (n=6). The data are presented as the mean ± standard error of the mean (SEM). A two-tailed Student’s t test was applied. **p<0.01, ***p<0.001, ****p<0.0001.

**Figure S2.Validation of cardiomyocyte-specific deletion of *Erk3*. a** Mouse tail genotyping of *Erk3*^flox/flox^*αMhc-MerCreMer*^Cre^ (*Erk3* cKO) mice. **b** IF of hearts from *Erk3* cKO and *Erk3*^flox/flox^ (Ctrl) mice. Red: ERK3; Yellow: alpha actinin (α-actin); Blue: 4',6-diamidino-2-phenylindole (DAPI). Scale bar: 20 μm.

**Figure S3. Cardiomyocyte-specific *Erk3* deficiency protects against pressure overload-induced cardiac dysfunction. a** Heart weight to tibia length ratio (HW/TL), left ventricle mass (LV Mass), left ventricular posterior wall dimension in diastole (LVPW d), left ventricular posterior wall thickness in systole (LVPW s), left ventricular internal dimension in diastole (LVID d), left ventricular Internal dimension in systole (LVID s) and left ventricular end-diastolic volume (LVEDV) of Ctrl and *Erk3* cKO mice after 4 weeks of TAC or sham operation (n=6 or 8 or 9). **b** Kapla-Meier survival curves of Ctrl and *Erk3* cKO mice challenged with TAC. **c** Length and width of adult mouse cardiomyocytes from Ctrl and *Erk3* cKO mice after 4 weeks of TAC or sham operation (n=6 or 8 or 9). Scale bar: 100 μm. The data are presented as the mean ± SEM. One-way ANOVA with the Tukey multiple comparison test was applied for **a** and **c**. Gehan–Breslow–Wilcoxon test was applied for **b**. *p<0.05, **p<0.01, ****p<0.0001, ns = not significant.

**Figure S4. Validation of *Erk3* overexpression and interference. a-b** Validation of *Erk3* overexpression plasmids (oe-*Erk3*) efficiency (n=5). **c-d** Validation of *Erk3* siRNA (si-*Erk3*) efficiency (n=5). The data are presented as the mean ± SEM. One-way ANOVA with the Dunnett multiple comparison test was applied for **c**. A two-tailed Student’s t test was applied for **b** and **d**. One-way ANOVA with the Tukey multiple comparison test was applied for **a**. **p<0.01, ***p<0.001, ****p<0.0001, ns = not significant.

**Figure S5.** *Erk3* **in the cardiomyocytes regulates myocardial cell function under pressure overload. a** The protein levels of atrial natriuretic peptide (ANP) and BNP in NRCMs transfected with the *Erk3* overexpression plasmids (oe-*Erk3*) or control plasmids (NC) and treated with or without Ang Ⅱ for 24 hours (n=5). **b** The protein levels of ANP and BNP in NRCMs transfected with si-*Erk3* or control siRNA (si-NC) and treated with or without Ang Ⅱ for 24 hours (n=5). The data are presented as the mean ± SEM. One-way ANOVA with the Tukey multiple comparison test was applied. **p<0.01, ***p<0.001, ****p<0.0001, ns = not significant.

**Figure S6. Validation of** *Aly* siRNA (si-*Aly*)**.** n=5. The data are presented as the mean ± SEM. One-way ANOVA with the Dunnett multiple comparison test was applied. **p<0.01, ***p<0.001, ns = not significant.

Figure S7. **Aly/REF export factor (ALY) mediates the function of ERK3. a** The protein levels of ANP and BNP in NRCMs transfected with the *Aly* overexpression plasmids (oe-*Aly*) or NC and treated with or without Ang Ⅱ for 24 hours (n=5). **b** The protein levels of ANP and BNP in Ang Ⅱ stimulated-NRCMs transfected with the si-*Erk3* or si-NC and oe-*Aly* or NC. The data are presented as the mean ± SEM. One-way ANOVA with the Tukey multiple comparison test was applied. **p<0.01, ***p<0.001, ****p<0.0001, ns = not significant.

**Figure S8. Estrone sulfate (ES) can relieve myocardial cell dysfunction through the ALY. a** The protein levels of ANP and BNP in Ang Ⅱ stimulated-NRCMs transfected with the si-*Aly* or si-NC and treated with or without ES for 24h (n=5). **b** The protein levels of ANP and BNP in Ang Ⅱ stimulated-NRCMs transfected with the si-*Erk3* or si-NC and treated with or without ES for 24h (n=5). The data are presented as the mean ± SEM. One-way ANOVA with the Dunnett multiple comparison test was applied. ***p<0.001, ns = not significant.

**Figure S9. ALY does not function as a RNA protein complex to regulate ERK3.** The protein levels of n-ERK3 and c-ERK3 in NRCMs transfected with the Aly without RNA Recognition Motif (△ALY) or NC and treated with Ang Ⅱ for 24h (n=6). The data are presented as the mean ± SEM. A two-tailed Student’s t test was applied. ****p<0.0001.

**Figure S10. ES can promote cardiac dysfunction under pressure overload. a** LV Mass, LVPW d, LVPW s, LVID d, LVID s, and LVEDV of mice treat with ES or not after 4 weeks of TAC operation (n=9). **b** The protein levels of ANP, BNP, collagen type I alpha 1 chain (COL1A1) and collagen type I alpha 1 chain (COL3A1) in heart tissues from mice treat with or without ES after 4 weeks of TAC operation (n=9). The data are presented as the mean ± SEM. A two-tailed Student’s t test was applied. *p<0.05, **p<0.01, ***p<0.001, ****p<0.0001.

**Figure S11. UMAP plot of snRNA-seq of hearts from hypertrophic cardiomyopathy patients and nonfailing humans.**

**Figure S12. ERK3 do not effect the protein levels of splicing factor proline and glutamine rich (SFPQ).** The protein levels of SFPQ in NRCMs transfected with the oe-*Erk3* or NC and treated with Ang Ⅱ for 24h (n=6). The data are presented as the mean ± SEM. A two-tailed Student’s t test was applied. ns = not significant.

**Figure S13. ERK3 regulate myocardial cell dysfunction through thioredoxin-interacting protein (TXNIP)** **in vitro. a** The protein levels of TXNIP and NLRP3 in NRCMs transfected with the oe-*Erk3* or NC and treated with or without Ang Ⅱ for 24h (n=5). **b** The protein levels of TXNIP and NOD-like receptor thermal protein domain associated protein 3 (NLRP3) in NRCMs transfected with the si-*Erk3* or si-NC and treated with or without Ang Ⅱ for 24h (n=5). **c** The protein levels of TXNIP and NLRP3 in Ang Ⅱ-treated NRCMs transfected with oe-*Aly* or NC and treated with or without SFPQ protein for 24h (n=5). **d** The protein levels of ANP and BNP in NRCMs transfected with the si-*Erk3* or si-NC and treated with or without SRI-37330 for 24h (n=5). **e** The protein levels of ANP and BNP in Ang Ⅱ stimulated-NRCMs transfected with the oe-*Erk3* or NC and treated with or without SRI-37330 for 24h (n=5). The data are presented as the mean ± SEM. One-way ANOVA with the Tukey multiple comparison test was applied. *p<0.05, **p<0.01, ***p<0.0001, ****p<0.0001, ns = not significant.

**Fig. S14. ERK3 regulate cardiac dysfunction and oxidative stress through TXNIP in vivo. a** HW/TL, LV Mass, LVPW d, LVPW s, LVID d, LVID s and LVEDV of Ctrl and *Erk3* cKO mice treat with SRI-37330 or not after 4 weeks of TAC operation (n=8). **b** NLRP3 immunofluorescence (IF) in heart tissues from Ctrl and *Erk3* cKO mice treat with SRI-37330 or not after 4 weeks of TAC operation (n=8). Scale bar: 50 μm. **c** Caspase-1 activity of hearts from Ctrl and *Erk3* cKO mice treat with SRI-37330 or not after 4 weeks of TAC operation (n=8). **d** IL-1β levels of hearts from Ctrl and *Erk3* cKO mice treat with SRI-37330 or not after 4 weeks of TAC operation (n=8). The data are presented as the mean ± SEM. One-way ANOVA with the Tukey multiple comparison test was applied. **p<0.01, ***p<0.0001, ****p<0.0001, ns = not significant.

| Target | Catalog no. | Host specie | Company | Dilution |
| --- | --- | --- | --- | --- |
| α-actin | 23660-1-AP | Rabbit | Proteintech | 1:200 for IF |
| ANP | PA3-228 | Rabbit | ThermoFisher | 1:1000 |
| BNP | PA5-98295 | Rabbit | ThermoFisher | 1:10000 |
| COL1A1 | 67288-1-Ig | Mouse | Proteintech | 1:5000 (1:2500 for IHC) |
| COL3A1 | 68320-1-Ig | Mouse | Proteintech | 1:1000 |
| ALY | Ab202894 | Rabbit | Abcam | 1:1000 (1:250 for IF,1:40 for IP) |
| Myc-Tag | 2276 | Rabbit | Cell Signaling Technology | 1:1000 (1:250 for IP) |
| GAPDH | 5174 | Rabbit | Cell Signaling Technology | 1:1000 |
| β-actin | 4970 | Rabbit | Cell Signaling Technology | 1:1000 |
| H3 | Ab1791 | Rabbit | Abcam | 1:1000 |
| ERK3 | SC-365234 | Mouse | Santa Cruz Biotechnology | 1:500 (1:100 for IF) |
| ERK3 | SC-374239 | Mouse | Santa Cruz Biotechnology | 1:500 (1:100 for IF,1:250 for IP, 5 μg for ChIP) |
| ERK3 | Ab53277 | Rabbit | Abcam | 1:1000 |
| TXNIP | 14715 | Rabbit | Cell Signaling Technology | 1:1000 |
| NLRP3 | 30109-1-AP | Rabbit | Proteintech | 1:1000 (1:200 for IF) |
| SFPQ | #71992 | Rabbit | Cell Signaling Technology | 1:1000 |
| SFPQ | Ab38148 | Rabbit | Abcam | 5 μg for ChIP |

**Table S1. Antibodies used**

| Gene name | Primer sequences | Species |
| --- | --- | --- |
| *Col1a1* | F: GCTCCTCTTAGGGGCCACT  R: CCACGTCTCACCATTGGGG | Mice |
| *Col3a1* | F: CTGTAACATGGAAACTGGGGAAA  R: CCATAGCTGAACTGAAAACCACC | Mice |
| *Erk3* | F: CCGAATTTGGTACACATGTGTT  R: CACCAGGACATTGATAAATGGC | Rat |
| *Erk3* | F: CACATGAACTTGAACAGATGCAG  R: CAGTGCTTCTCGGCTGATCC | Mice |
| *Txnip* | F: GCTCAATCATGGTGATGT TCAAG  R: CTTCACACACTTCCACTGTCAC | Rat |
| *Bnp* | F:GGACCAAGGCCCTACAAAAGAACT  R: CTAAAACAACCTCAGCCCGTCACA | Rat |
| *Txnip*-1(ChIP) | F: CTGATGGAGGCACAGTGAGA  R: CTCGGGTGGAGTGCTTAGAG | Rat |
| *Txnip*-2(ChIP) | F: TACAGGTGAGAACGAGATGGTGA  R: TTGAGTTGGCTGGCTGGGAC | Rat |
| *Txnip*-3(ChIP) | F: GGCTTTTCTCGATCGTCCCA  R: AGAGGCAGAAAGCGTTGAGT | Rat |
| *αMHC-MerCreMer* (mouse tail genotyping) | F: TCTATTGCACACAGCAATCCA  R: CCAGCATTGTGAGAACAAGG | Mice |
| Wildtype (mouse tail genotyping) | F: TCTATTGCACACAGCAATCCA  R: CCAACTCTTGTGAGAGGAGCA | Mice |
| *Erk3* (mouse tail genotyping) | F: ATTCCCTGGTCACTGATGAAGTC  R:CCTTAACACTCTCCAGTTACTGAGC | Mice |

Table S2.**Oligonucleotides used for PCR**

| Gene name | siRNA sequences | Species |
| --- | --- | --- |
| *Erk3*-1 | F: CGAUGAAGAAGAAGUUCAAGU  R: UUGAACUUCUUCUUCAUCGGU | Rat |
| *Erk3*-2 | F: GGAGGAUUCUGAAAUGCUAGA  R: UAGCAUUUCAGAAUCCUCCUU | Rat |
| *Erk3*-3 | F: GACGUAGUUGACAAGUUAAAC  R: UUAACUUGUCAACUACGUCAG | Rat |
| *Aly*-1 | F: GGAAGCUGCUUGUGUCAAACC  R: UUUGACACAAGCAGCUUCCCA | Rat |
| *Aly*-2 | F: GCUGGAUGCUUACAAUGCAAG  R: UGCAUUGUAAGCAUCCAGCUG | Rat |
| *Aly*-3 | F: GGAGUGUCAGACGCUGAUAUU  R: UAUCAGCGUCUGACACUCCGA | Rat |

**Table S3. siRNA used**

| Proteins | ratio | Proteins | ratio | Intersection |
| --- | --- | --- | --- | --- |
| H1F2 | 23.55450237 | H1F4 | 18.93389173 | RBM26 |
| ACTA2 | 19.83800116 | RBM26 | 14.06043365 | YBX3 |
| CSNK1D | 19.52539305 | FTSJ3 | 13.15190131 | TCOF1 |
| TCOF1 | 16.52906628 | YBX3 | 11.85761817 | MYBBP1A |
| KRT42 | 13.50921415 | TCOF1 | 10.58625745 | MYO18A |
| CXCL12 | 11.66640871 | Mybbp1a | 10.1264365 | POLDIP3 |
| ARL8B | 8.852527007 | CDIPT | 9.111152553 | TMEM33 |
| EWSR1 | 8.064860587 | DHX9 | 7.136049982 | SRP9 |
| PPIB | 7.115384615 | MYO18A | 6.8258666 | ALYREF |
| DAXX | 6.429072605 | POLDIP3 | 6.726381193 | GTPBP4 |
| PPP1CA | 6.363675869 | KHDRBSL | 5.751607231 | KRT42 |
| FAM98A | 5.784413961 | ELAVLA1 | 4.87599887 | KRT75 |
| NHERF2 | 5.74895642 | TMEM33 | 4.580904828 | FUS |
| SRP9 | 5.641222521 | MYL4 | 4.573715018 | PES1 |
| FUS | 5.552301503 | SSB | 4.549288682 | GOT1 |
| ALYREF | 5.488764045 | COPA | 4.401577258 | NAT10 |
| RBM26 | 5.299929145 | HNRNPC | 4.38259039 | RTRAF |
| LSM12 | 5.210215443 | SRP9 | 4.303254381 | CDC5l |
| PES1 | 4.994667453 | ALYREF | 4.283261803 | SFPQ |
| POLDIP3 | 4.97454813 | Ccdc80 | 4.272715245 | DAXX |
| YBX1 | 4.827701069 | UPF1 | 4.003190407 |  |
| NAT10 | 4.805382181 | GTPBP4 | 3.888895354 |  |
| EZR | 4.751173491 | KRT42 | 3.864168618 |  |
| NOB1 | 4.725014883 | EPHB4 | 3.701700028 |  |
| KRT75 | 4.692998776 | LTV1 | 3.442725508 |  |
| GOT1 | 4.619522214 | NF2 | 3.438568288 |  |
| GTPBP4 | 4.611655729 | EIF2S1 | 3.390246242 |  |
| MYO18A | 4.509031406 | KRT75 | 3.282833986 |  |
| MYBBP1A | 4.260866003 | DDX47 | 3.155825524 |  |
| TMEM33 | 3.853742162 | TMPRSS13 | 3.133971292 |  |
| LLPH | 3.771399141 | DDX1 | 3.129990868 |  |
| ARPC5 | 3.697139972 | FUS | 3.022089898 |  |
| CYC1 | 3.614733308 | LRRC59 | 2.957693136 |  |
| RDX | 3.540699248 | RBMXL1B | 2.915095171 |  |
| SFPQ | 3.423728276 | PES1 | 2.875923227 |  |
| JUP | 3.370706574 | GOT1 | 2.871692402 |  |
| PURA | 3.108683565 | RBM14 | 2.858556145 |  |
| KRT16 | 3.056270183 | EIF1A | 2.845864938 |  |
| FARP1 | 3.030231223 | NAT10 | 2.76980274 |  |
| AP2A1 | 2.99219887 | DIMT1 | 2.7010208 |  |
| CDC5L | 2.902686515 | PLEKHF1 | 2.700835737 |  |
| EPB41L3 | 2.892884331 | PABPC4 | 2.674244847 |  |
| HILS1 | 2.884479856 | RTRAF | 2.666179994 |  |
| Fn1 | 2.865234751 | Rtcb | 2.59991313 |  |
| Hadhb | 2.843023256 | Tmem109 | 2.599868551 |  |
| Ybx3 | 2.65490944 | CDC5l | 2.595914484 |  |
| Ddx21 | 2.644029319 | MYL9 | 2.544317135 |  |
| Rtraf | 2.58493924 | SFPQ | 2.509251997 |  |
| Fen1 | 2.573176826 | Daxx | 2.456881056 |  |
| Krt15 | 2.541618041 | Mrpl46 | 2.449918 |  |

**Table S4. The top 50 proteins with the highest changes in two CoIP/LC-MS**
